# Supplementary material for: A comprehensive examination of Nanopore native RNA sequencing for characterization of complex transcriptomes
Source: Nat Commun. 2019 Jul 31;10:3359. doi: 10.1038/s41467-019-11272-z (PMC6668388; doi:10.1038/s41467-019-11272-z)
Supplement: Supplementary file 1 — Supplementary Information [file 41467_2019_11272_MOESM1_ESM.pdf]

A comprehensive examination of Nanopore native RNA sequencing for characterization of complex transcriptomes

Soneson et al.

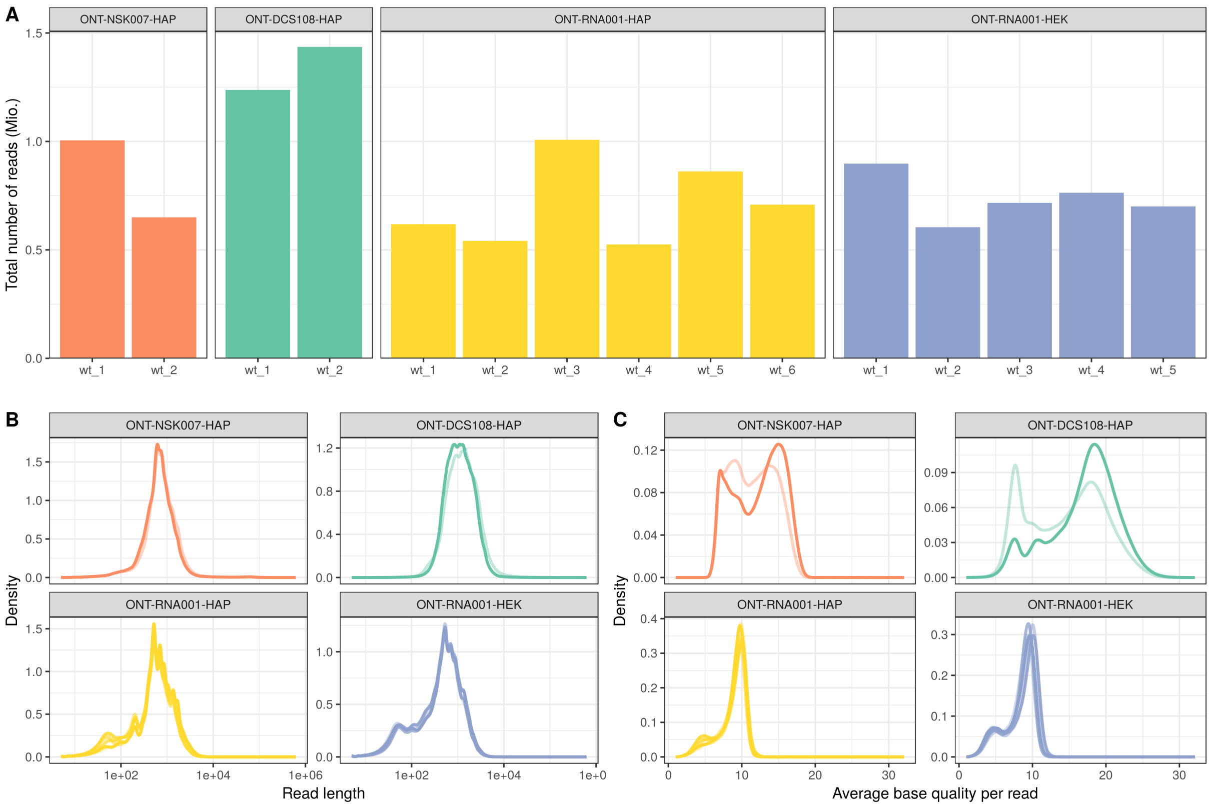

Supplementary Figure 1: Summary of the full set of reads in each ONT library, stratified by data set. A. Total number of reads. B. Read length distribution. C. Average base quality distribution. In B-C, each line corresponds to one library. Source data for panel A are provided as a Source Data file.

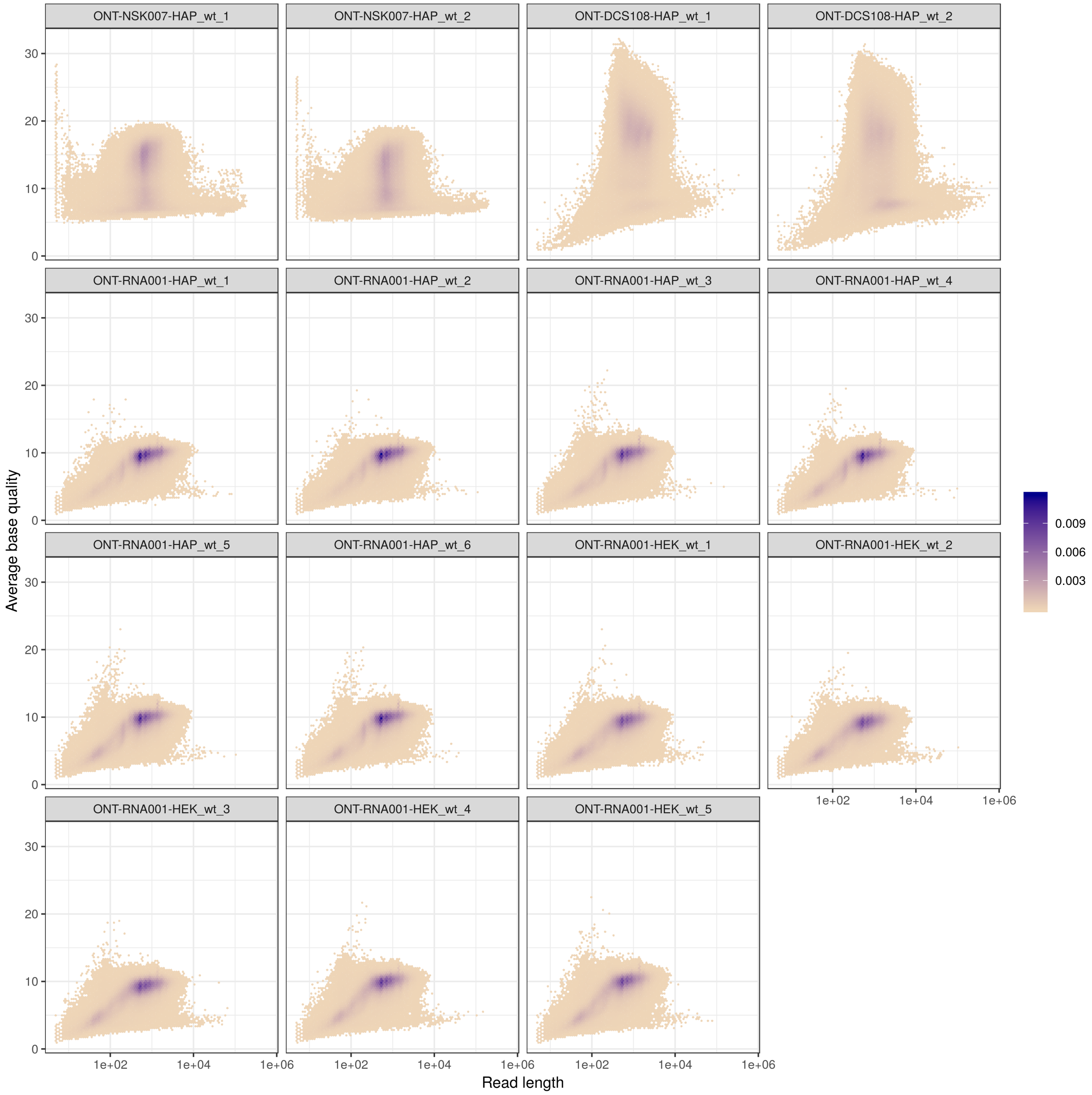

Supplementary Figure 2: Total read length (x) vs average base quality (y, as reported in the basecalled FASTQ file), for all reads in each of the ONT libraries. The colour indicates point density.

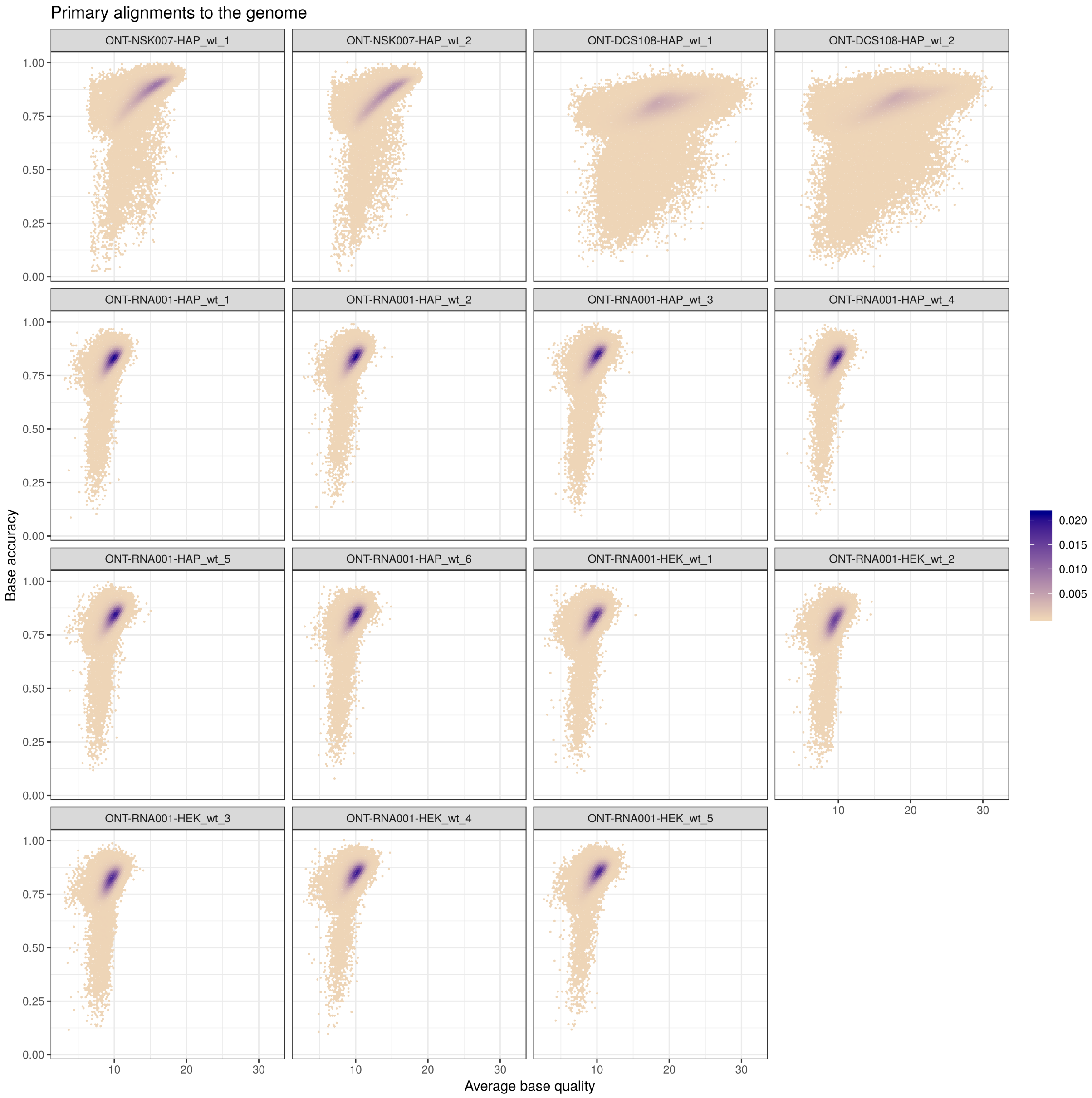

Supplementary Figure 3: Average base quality (x, as reported in the basecalled FASTQ file) and base-level accuracy (y, calculated as $(nbrM + nbrI + nbrD - NM)/(nbrM + nbrI + nbrD)$ from the part of the read involved in the primary genome alignment), for all aligned reads in each of the ONT libraries. Here, $nbrM$, $nbrI$ and $nbrD$ are the reported number of M, I and D characters in the CIGAR string of the alignment, and NM is the reported edit distance. The colour indicates point density. The mean accuracies were 85% (ONT-NSK007-HAP), 80% (ONT-DCS108-HAP), 83% (ONT-RNA001-HAP) and 83% (ONT-RNA001-HEK), respectively.

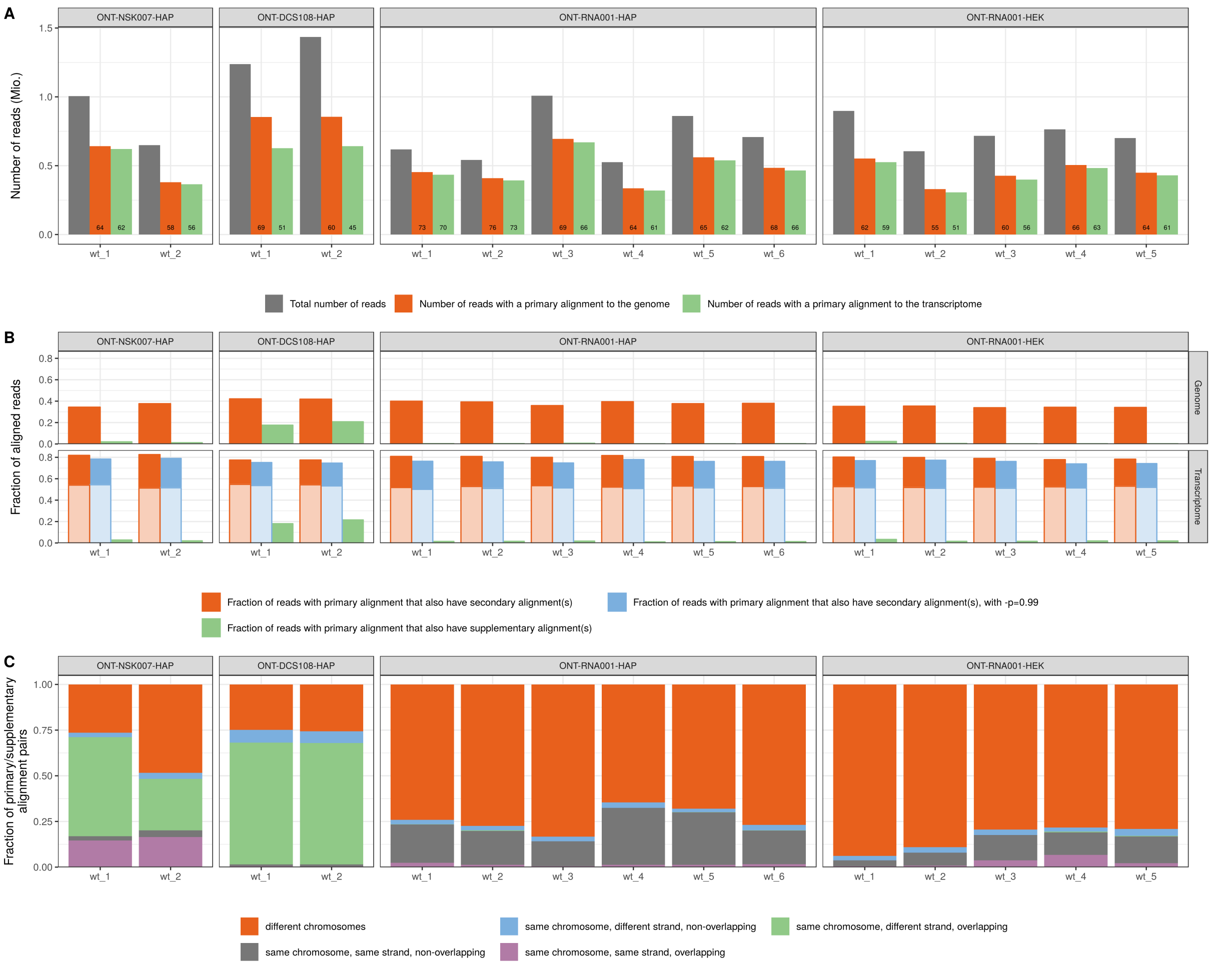

Supplementary Figure 4: Characterization of aligned reads in each of the ONT libraries. A. Total number of reads and the number of reads with a primary alignment to the genome or transcriptome, respectively. The number displayed in each bar represents the alignment rate in % (the fraction of the total number of reads for which minimap2 reports a primary alignment). B. Fraction of the reads with a primary alignment to the genome or transcriptome, respectively, that also have at least one reported secondary or supplementary alignment. The lighter shaded parts of the secondary transcriptome alignment bars correspond to reads where all primary and secondary alignments are to isoforms of the same gene, while the darker shaded parts correspond to reads with reported alignments to transcripts from different genes. C. Investigation of supplementary genome alignments. Each supplementary alignment is categorized based on whether it is on the same chromosome and strand as the primary alignment, and if the alignment positions of the primary and supplementary alignments overlap. Source data are provided as a Source Data file.

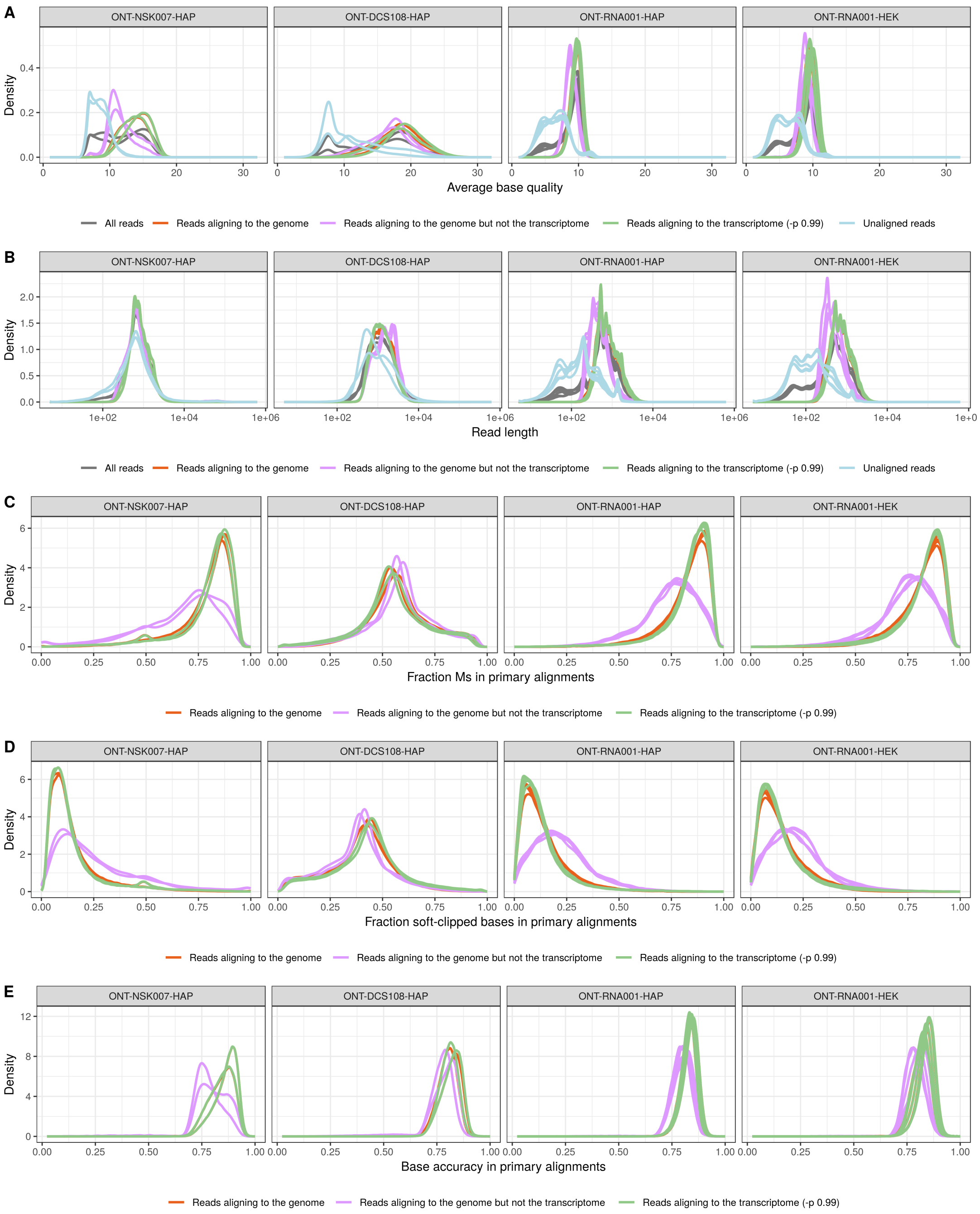

Supplementary Figure 5: Summary of read properties by alignment status. Each density corresponds to one ONT library. A. Average base quality distribution. Unaligned reads are predominantly found among reads with low base quality. B. Read length distribution. C. Number of M characters in the CIGAR string of the primary alignment to the genome and transcriptome, respectively, divided by the read length. D. Number of soft-clipped (S) bases in the CIGAR string of the primary alignment to the genome and transcriptome, respectively, divided by the read length. E. Base accuracy, calculated as $(nbrM + nbrI + nbrD - NM)/(nbrM + nbrI + nbrD)$ from the part of the read involved in the primary alignment.

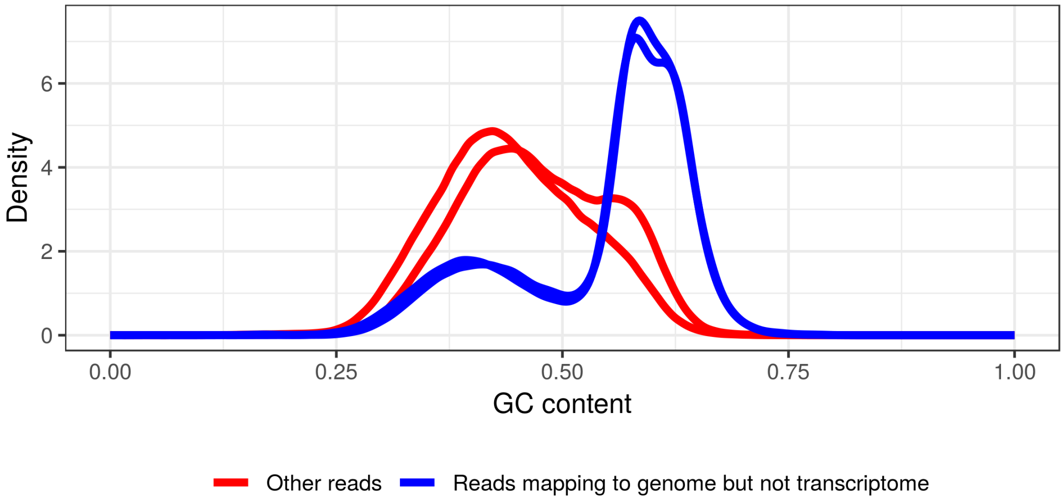

Supplementary Figure 6: GC content distribution for reads mapping to the genome but not the transcriptome, and all other reads, in the two ONT-DCS108-HAP libraries.

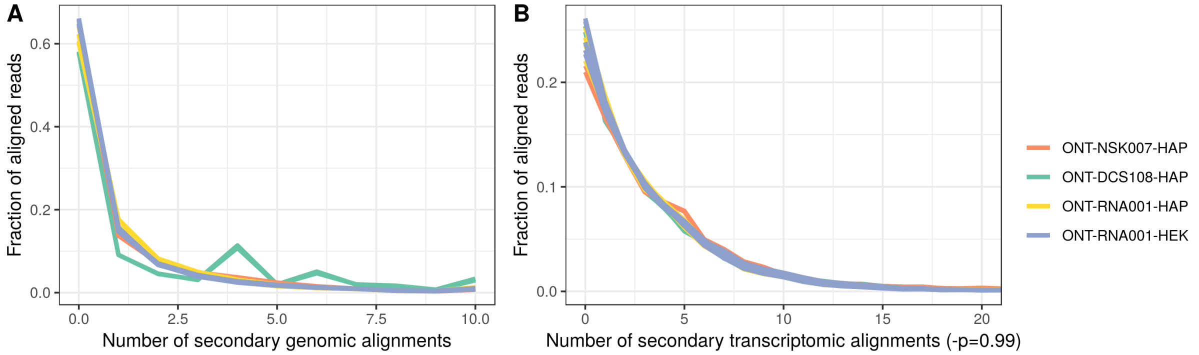

Supplementary Figure 7: Distribution of the number of secondary genome and transcriptome (with the `-p 0.99` setting) minimap2 alignments, for each ONT library. A. For the genome alignments, up to 10 secondary alignments were allowed. B. For the transcriptome alignments, up to 100 secondary alignments were allowed, in order to allow for the high similarity among isoforms. However, very few reads had more than 20 secondary alignments, and thus the x-axis is truncated to the range [0-20]. Source data are provided as a Source Data file.

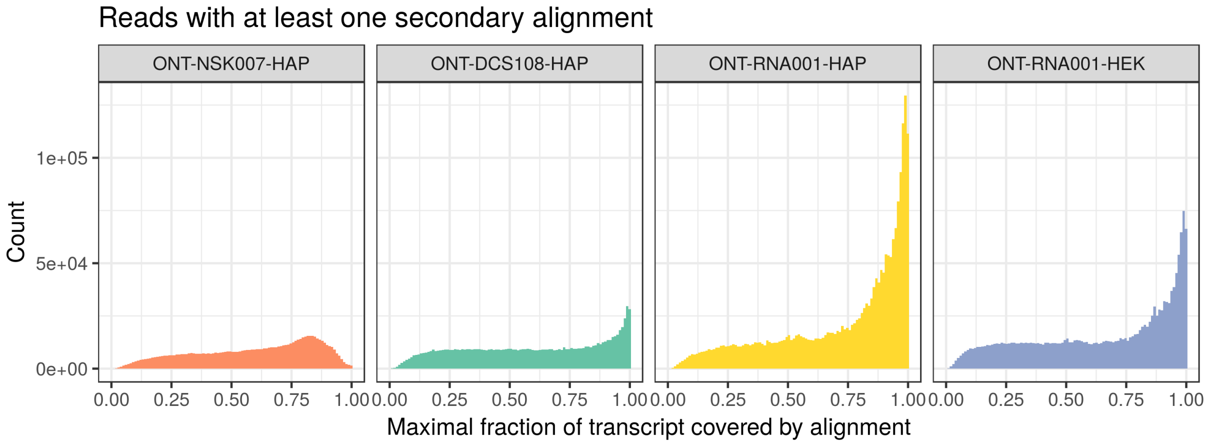

Supplementary Figure 8: The highest degree of coverage of a reference transcript by single ONT reads, for each read with at least one secondary alignment.

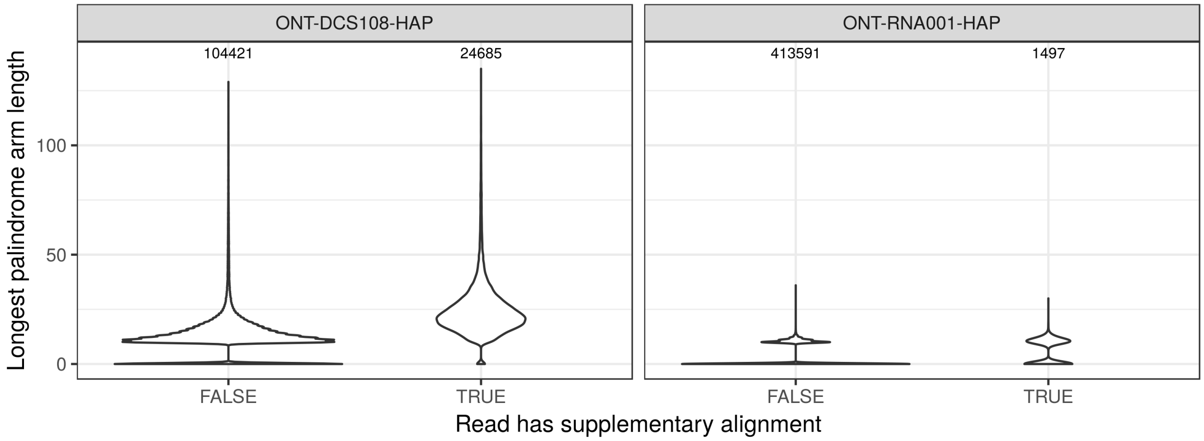

Supplementary Figure 9: Distribution of the length of the longest palindrome found in aligned reads with and without supplementary alignments, in the ONT-DCS108-HAP and ONT-RNA001-HAP libraries, aggregated by data set. For each library, 100,000 reads were randomly selected; only the ones with reported alignments are considered here. Only palindromes with arm length exceeding 10 bases were considered, reads without such palindromes were assigned a maximal palindrome arm length of 0.

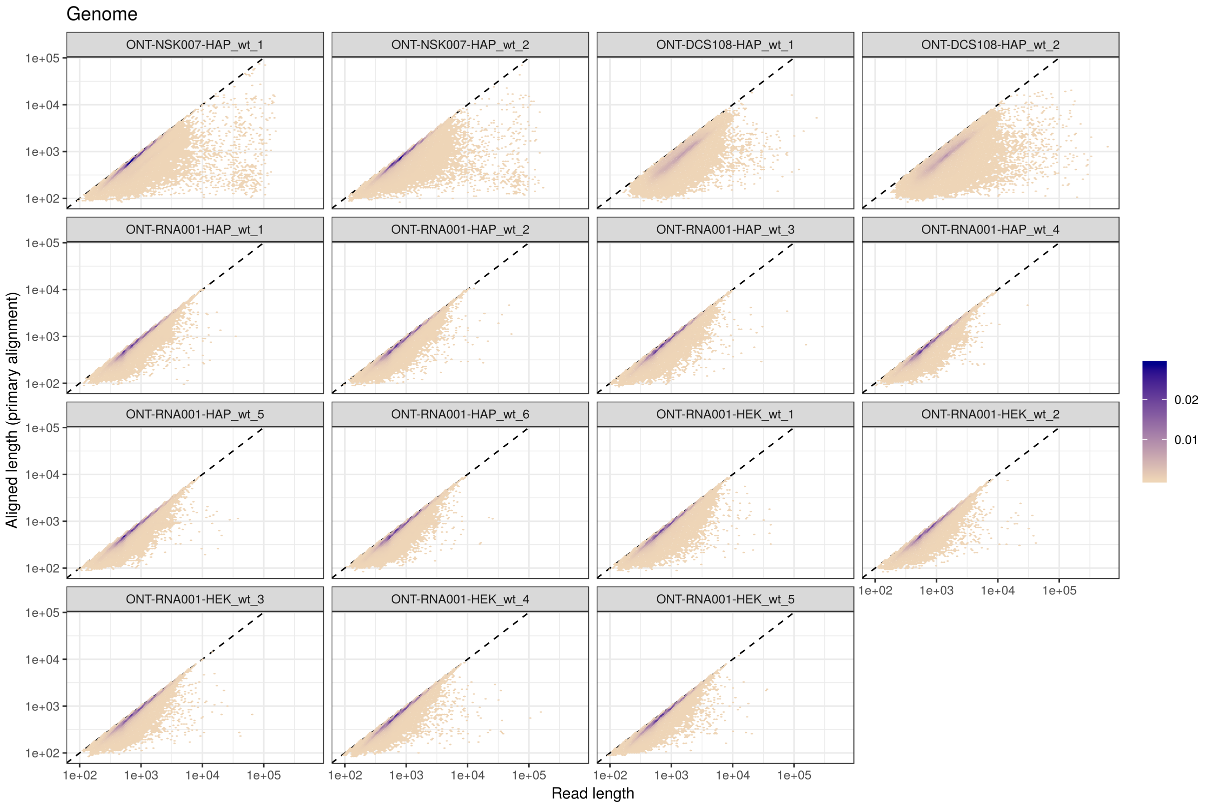

Supplementary Figure 10: Total read length (x) vs aligned length (y, the sum of the number of M and I characters in the CIGAR string) for the primary genome alignment of each read in each of the ONT libraries. The colour indicates point density.

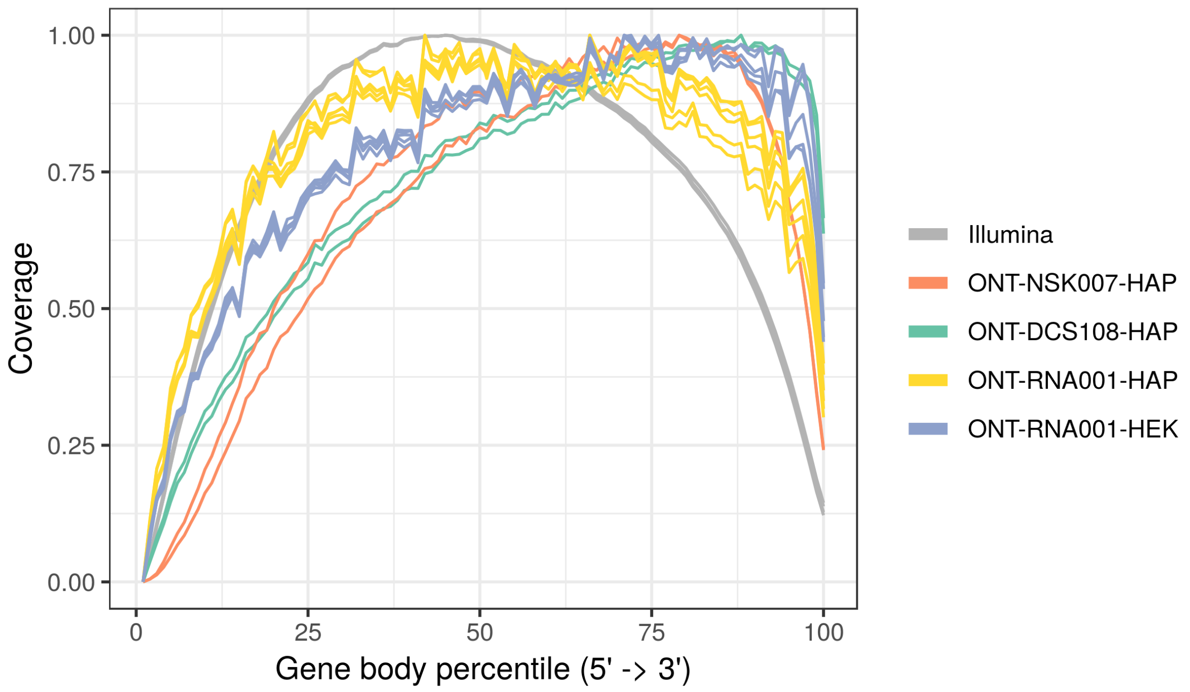

Supplementary Figure 11: Gene body coverage, estimated by RSeQC, in the ONT and Illumina libraries.

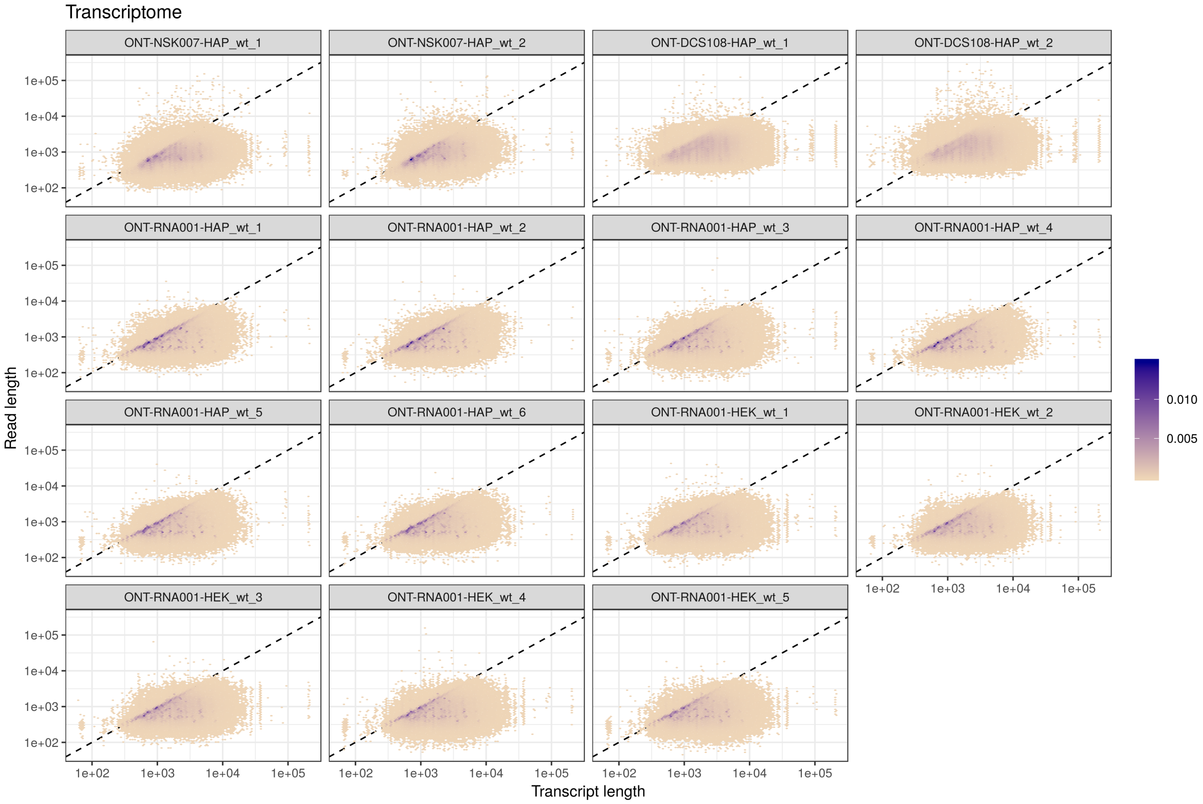

Supplementary Figure 12: Annotated length of the target transcript (x) vs total read length (y) for all primary transcriptome alignments in each of the ONT libraries. Reads aligning to the shortest transcripts are often longer than the transcript, and are thus soft clipped in the alignment. The colour indicates point density.

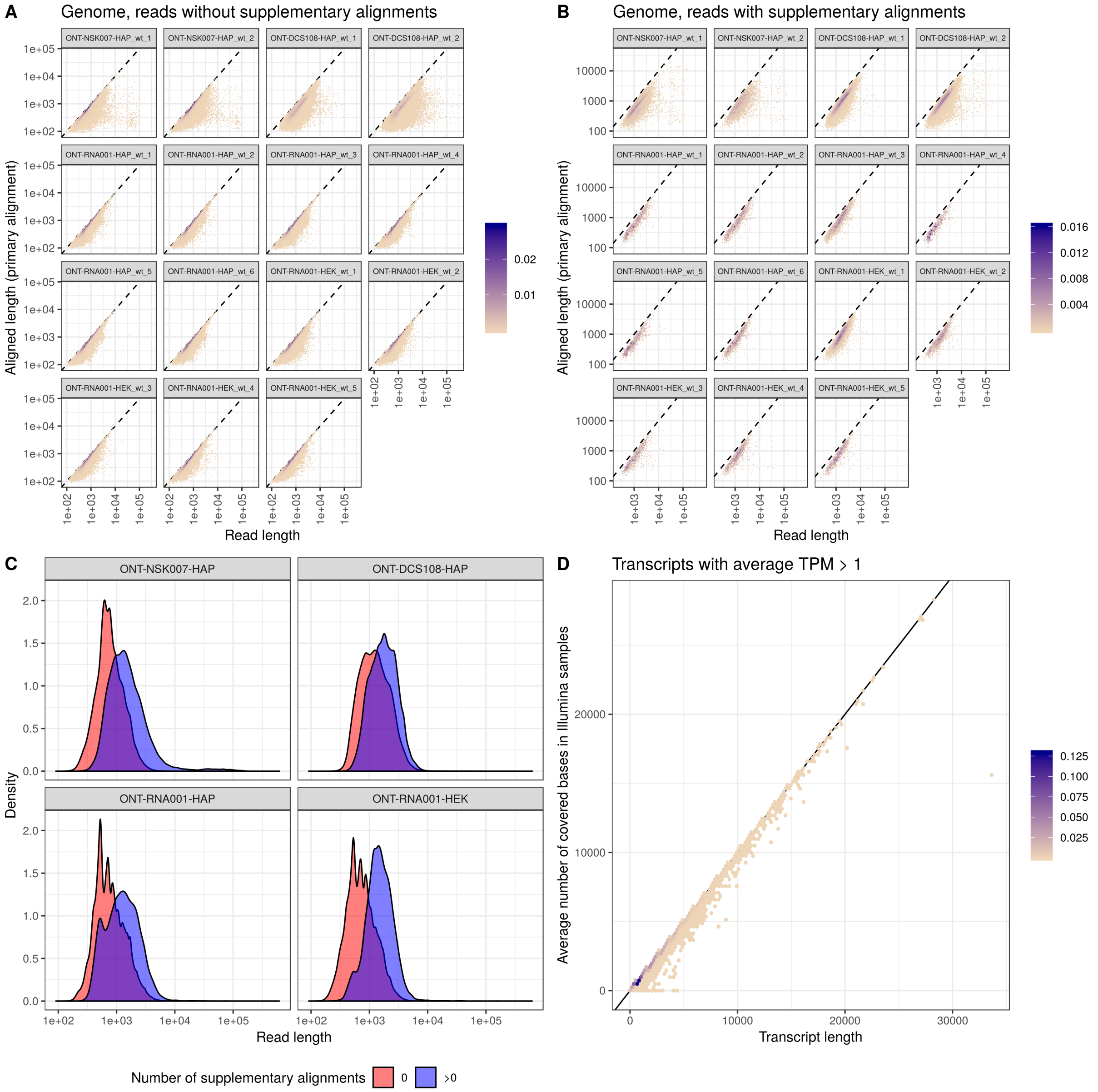

Supplementary Figure 13: A-B. Total read length (x) vs length of the primary genome alignment (y) for reads without (A) and with (B) any reported supplementary alignment. The colour indicates point density. C. Read length distribution for reads without (red) and with (blue) any reported supplementary alignment, in the four ONT data sets. D. The average number of nucleotides annotated to the transcript that are covered by reads in the Illumina samples *vs* the transcript length, for transcripts with an average estimated TPM exceeding 1 across the Illumina samples.

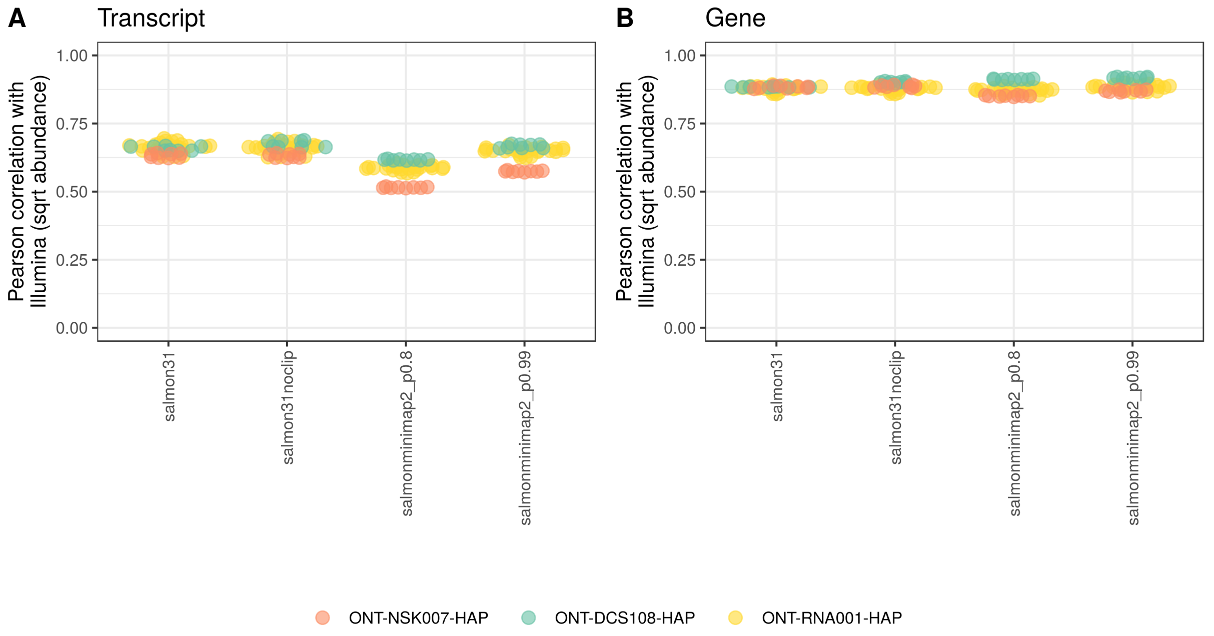

Supplementary Figure 14: Correlation between estimated abundances in the Illumina samples and those obtained from the ONT data, running Salmon in different configurations. ONT abundances are estimated counts, and Illumina abundances are estimated TPMs from Salmon. Each point corresponds to a pair of one Illumina sample and one ONT sample, and all such pairwise combinations were considered. Source data are provided as a Source Data file.

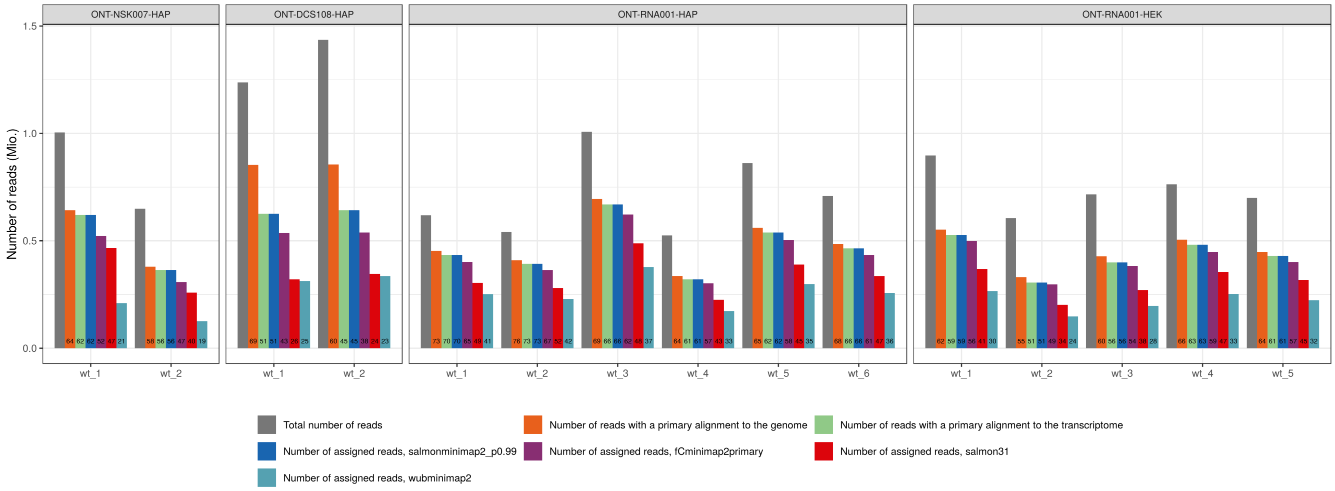

Supplementary Figure 15: Number of reads assigned to features (genes or transcripts) by each of the abundance estimation methods, for each ONT library. The number written in each bar indicates the percentage of the total number of reads that are assigned to features by the corresponding method. Source data are provided as a Source Data file.

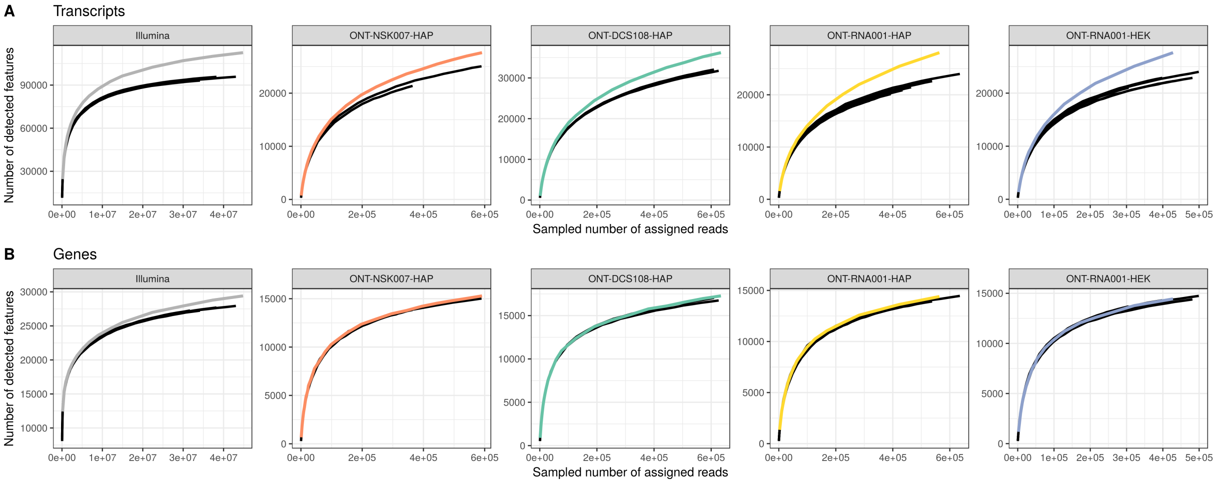

Supplementary Figure 16: Saturation curves for transcript (A) and gene (B) detection. Black curves represent individual libraries, and colored curves are obtained by pooling the reads across all samples in the data set before subsampling. These curves are truncated to the same range as the individual sample curves to facilitate comparison. A feature is considered detected if it has an estimated salmonminimap2 count (ONT libraries) or Salmon count (Illumina libraries) ≥ 1.

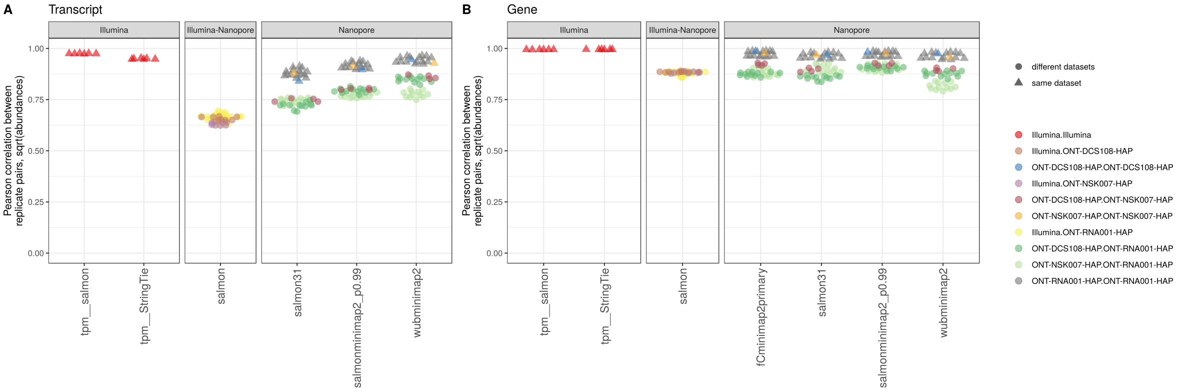

Supplementary Figure 17: Pearson correlations between transcript (A) or gene (B) abundances for each pair of samples, within and between data sets, for each abundance estimation method. Triangles correspond to pairs of samples within the same data set, and circles to pairs from different data sets. Correlations were calculated between square root-transformed estimated counts from the respective ONT methods, and for square root-transformed estimated TPMs for the Illumina libraries. Source Data are provided as a Source Data file.

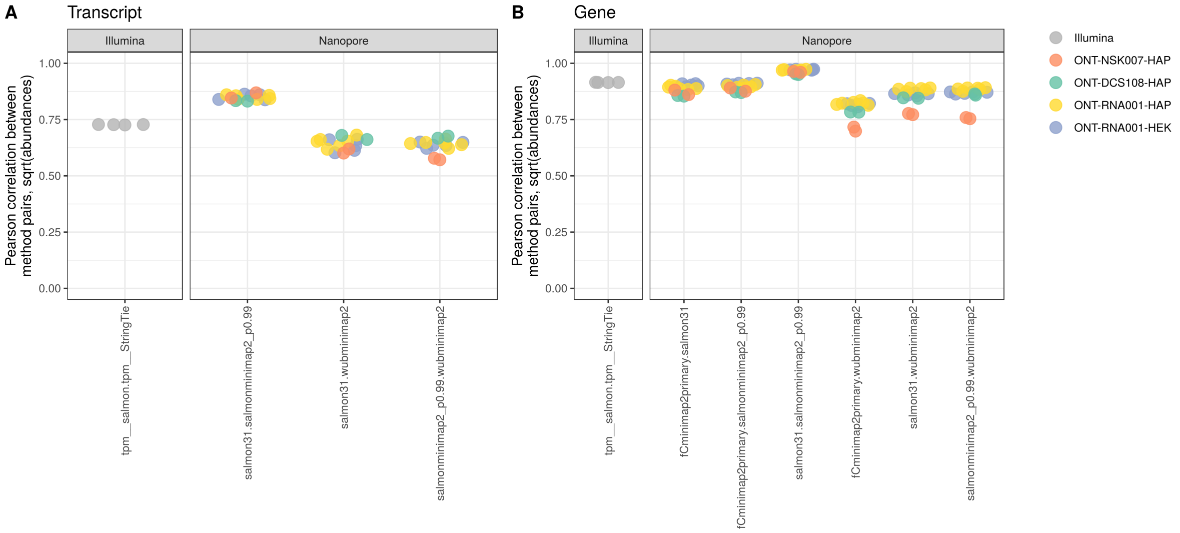

Supplementary Figure 18: Pearson correlations between transcript (A) or gene (B) abundances for each pair of abundance estimation methods, for each library. Correlations were calculated between square root-transformed estimated counts from the respective ONT methods, and for square root-transformed estimated TPMs for the Illumina libraries. Source data are provided as a Source Data file.

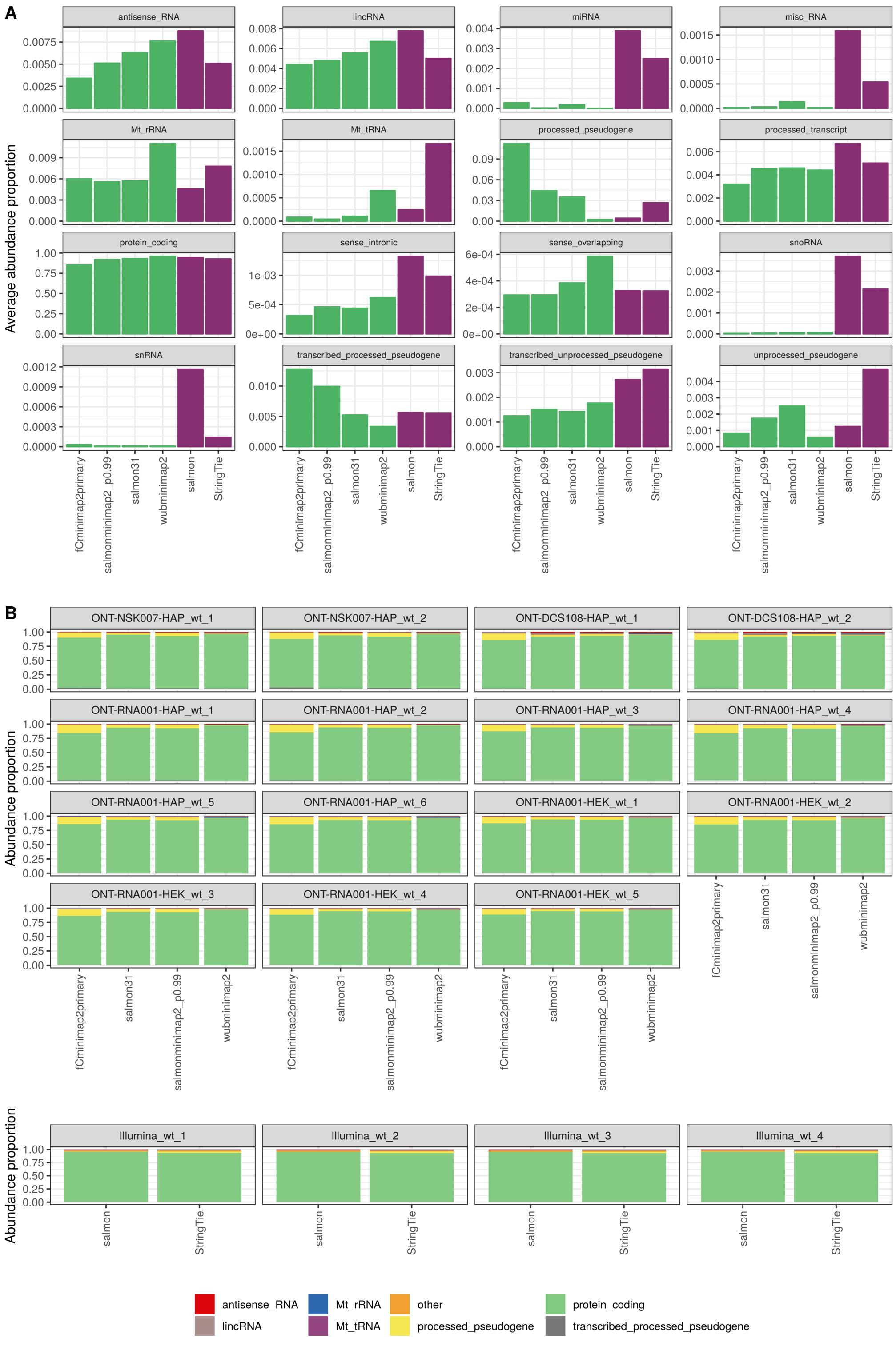

Supplementary Figure 19: A. Relative abundance (proportion of total abundance) assigned to genes of different biotypes by the different quantification methods, averaged across all libraries. Green bars represent abundances (counts) for ONT data, while purple bars represent abundances (TPMs) for the Illumina libraries. B. Relative abundance (proportion of total count) assigned to genes of different biotypes by the different quantification methods, for all libraries. Only the most abundant biotypes are represented separately, all others are collapsed into the ’other’ category. Source data are provided as a Source Data file.

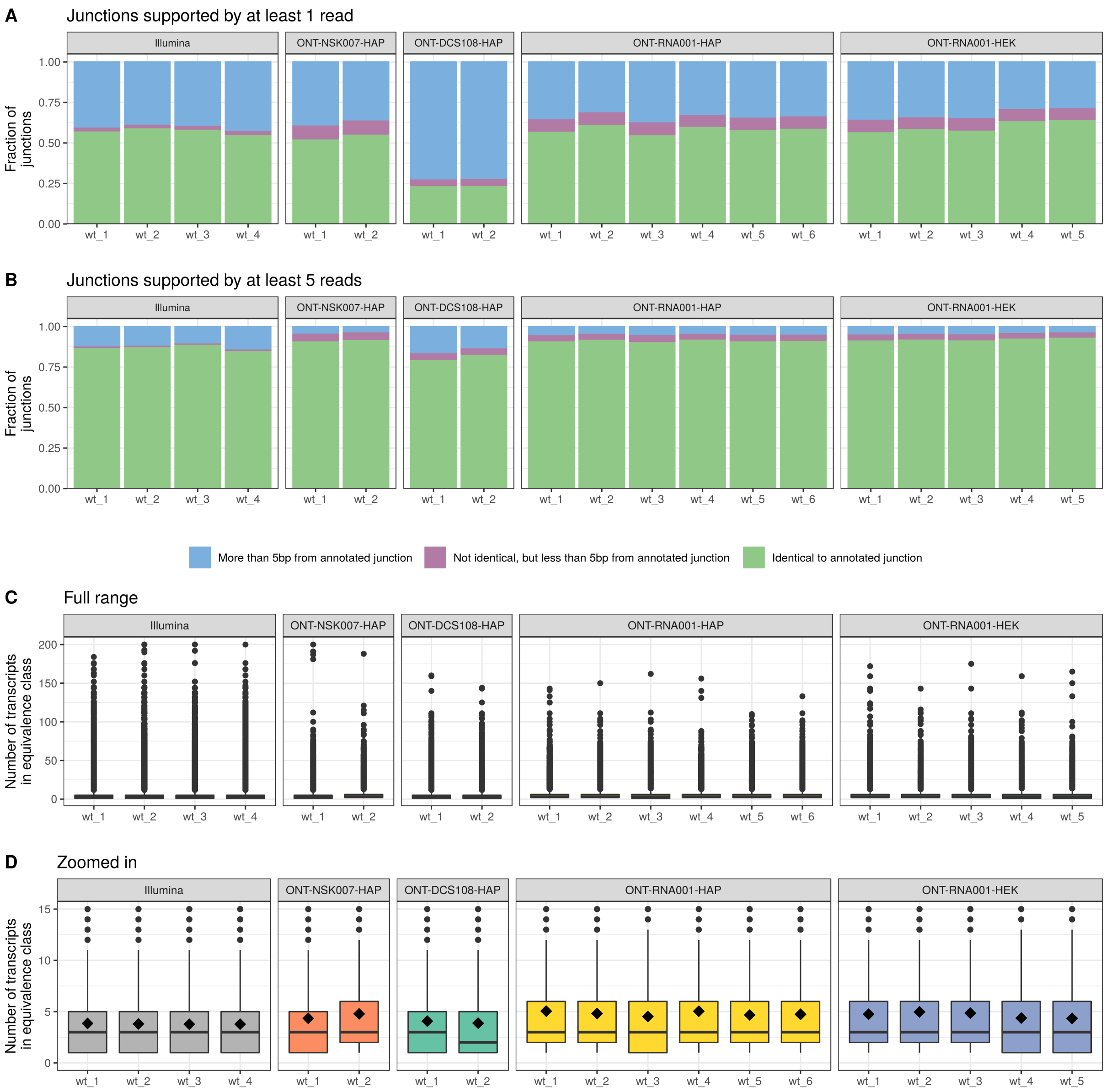

Supplementary Figure 20: A-B. Annotation status of junctions observed in each ONT and Illumina library. A junction is considered observed if it is supported by at least 1 (A) or 5 (B) reads. For each observed junction, the distance to each annotated junction was defined as the absolute difference between the start positions plus the absolute difference between the end positions. This distance was used to find the closest annotated junction. C. Distribution of the number of transcripts contained in the Salmon equivalence class that a read is assigned to, across all reads, for each ONT and Illumina library. D. As C, but zoomed in to the range [0, 15]. The black diamond shape indicates the mean. The center line represents the median; hinges represent first and third quartiles; whiskers the most extreme values within 1.5 interquartile range from the box. Source data are provided as a Source Data file.

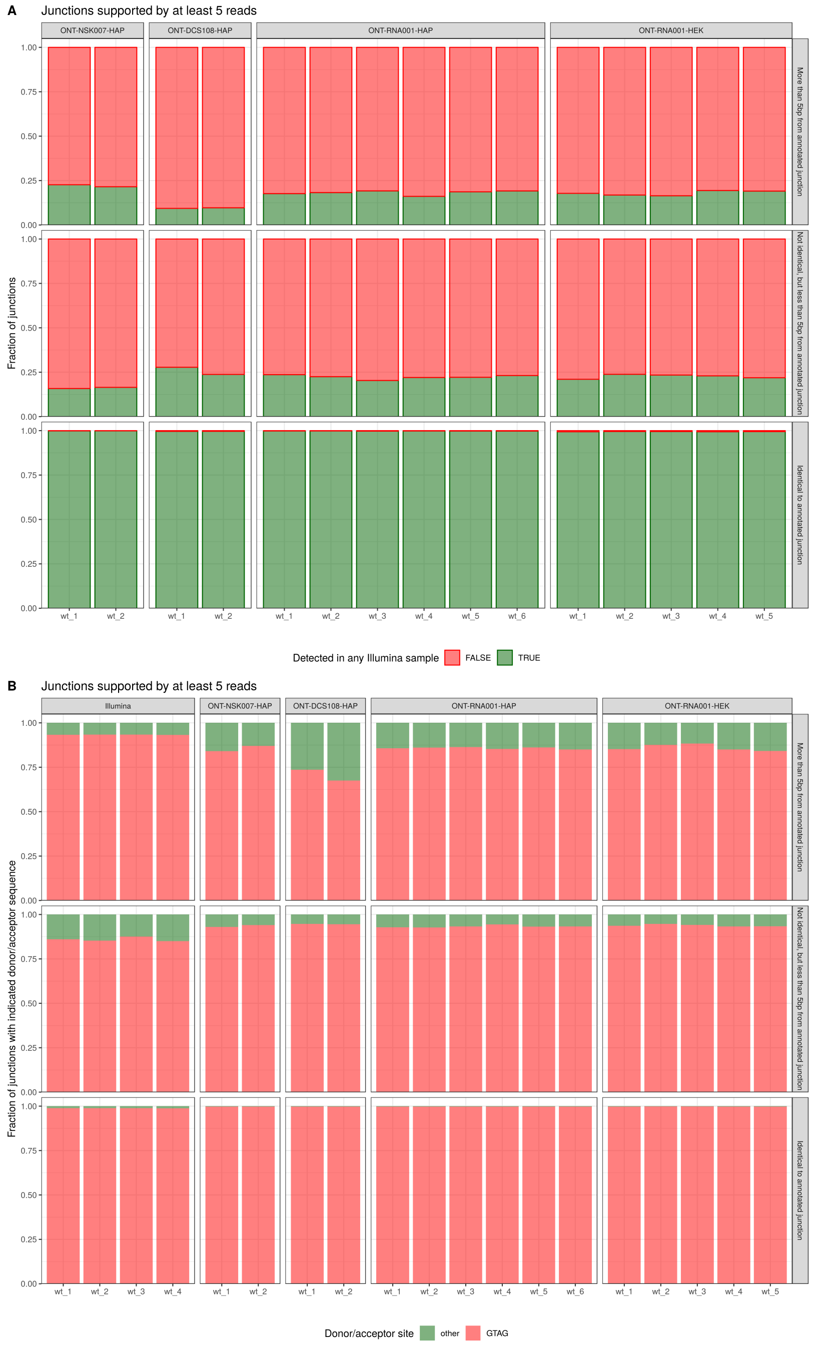

Supplementary Figure 21: A. Fraction of the junctions covered by at least 5 ONT reads that are also detected in at least one of the Illumina samples (supported by at least 1 read), for each category. B. Fraction of the junctions that contain a canonical (GT-AG) or non-canonical splicing motif, respectively, for each category. Source data are provided as a Source Data file.

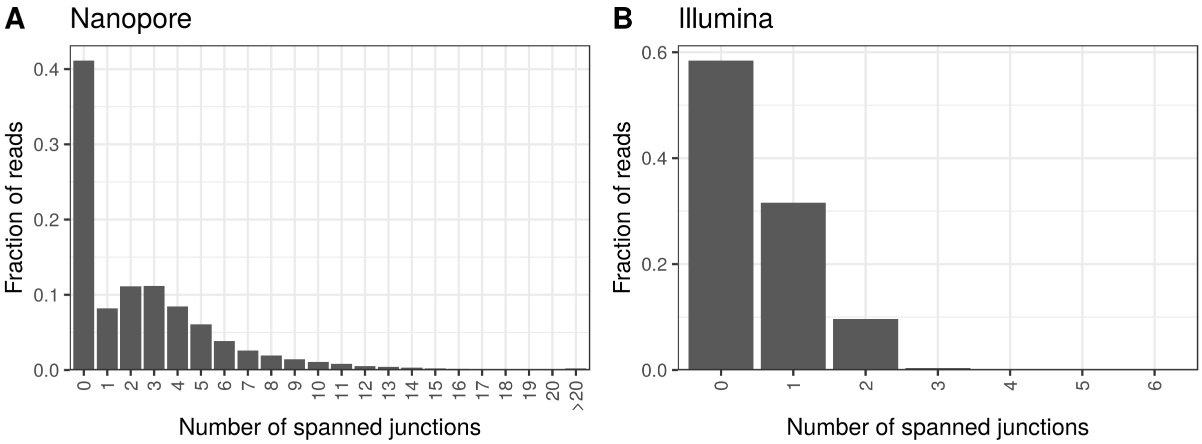

Supplementary Figure 22: Distribution of the number of junctions spanned by individual reads in the ONT (A) and Illumina (B) libraries. For each Illumina library, the number of spanned junctions were counted for a subset of 5 million reads, and only reads being the first in a properly mapped pair were contributing to the summary shown in this plot. Source data are provided as a Source Data file.

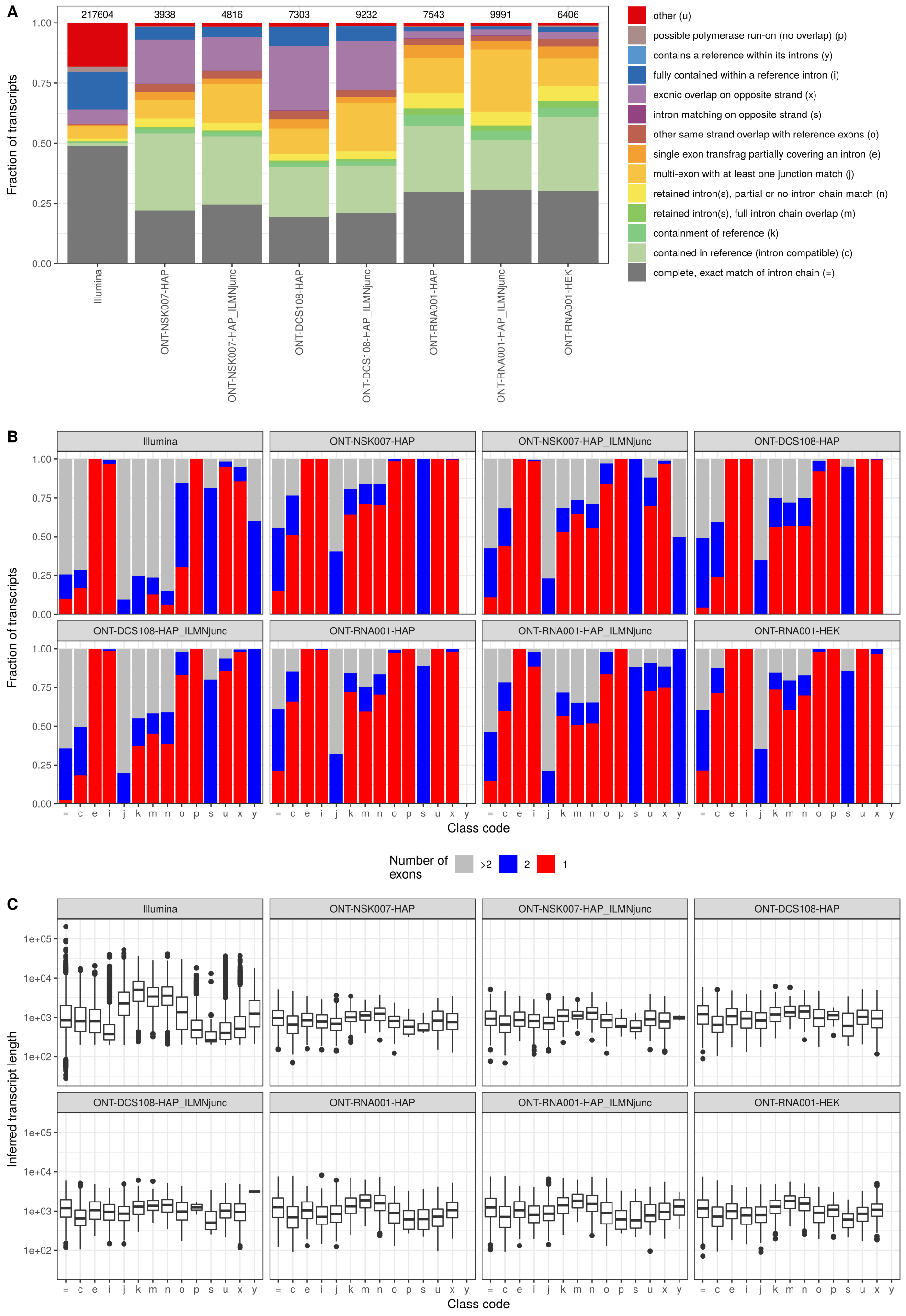

Supplementary Figure 23: Characterization of transcripts identified by FLAIR. A. Class code distribution for de novo identified transcripts from FLAIR (for ONT libraries) or StringTie (for Illumina libraries), compared to the set of annotated transcripts using gffcompare. The number above each bar represents the number of assembled transcripts. The class code of a transcript indicates its relation to the closest annotated transcript. B. Number of exons in each transcript identified by FLAIR/StringTie, stratified by the relation to the annotated transcripts (represented by the assigned class code). C. Length distribution of transcripts identified by FLAIR/StringTie, stratified by the relation to the annotated transcripts (represented by the assigned class code). The *_ILMNjunc* suffix indicates that FLAIR was supplied with the junctions observed in the Illumina samples. The center line represents the median; hinges represent first and third quartiles; whiskers the most extreme values within 1.5 interquartile range from the box. Source data for panels A-B are provided as a Source Data file.

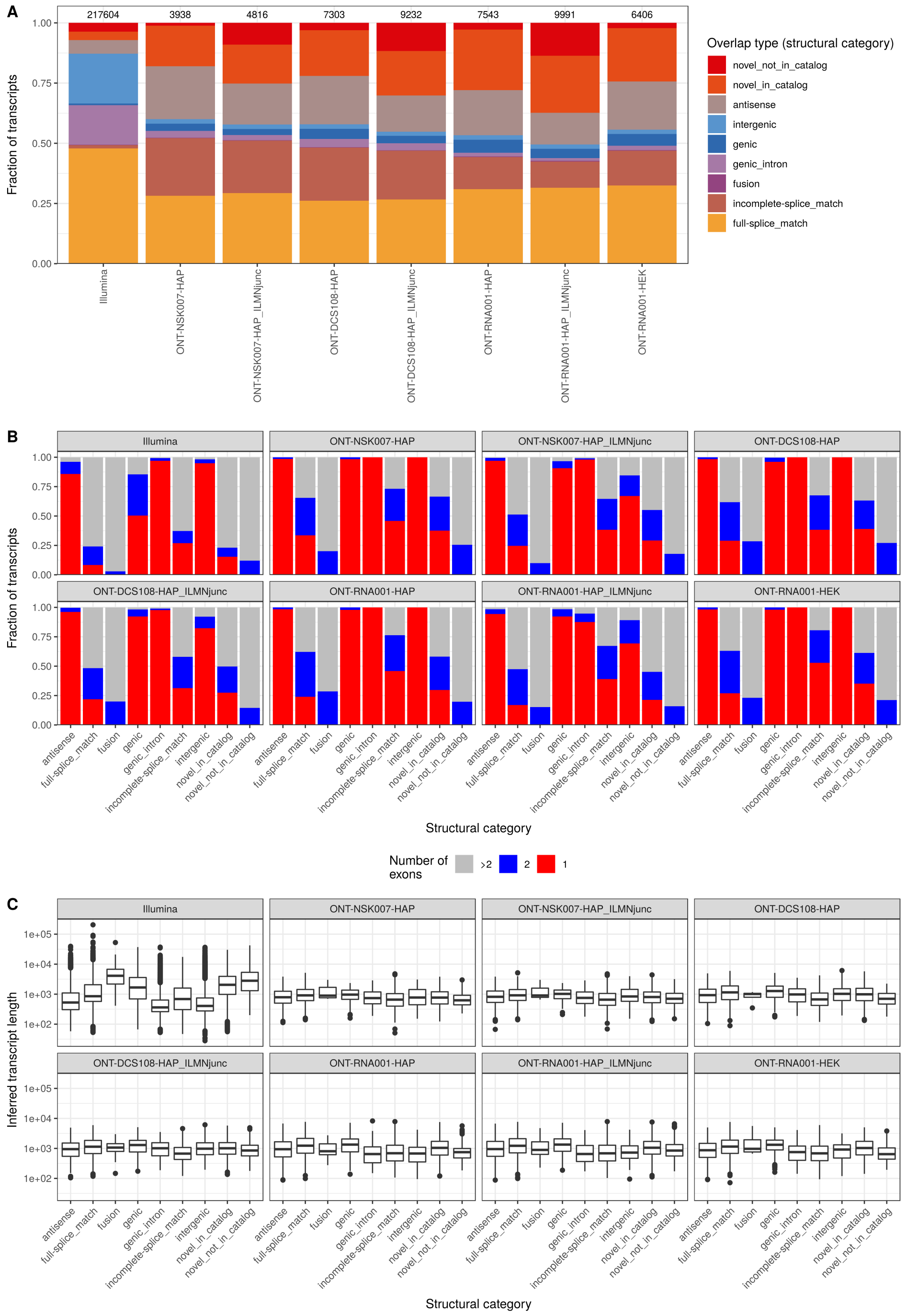

Supplementary Figure 24: Characterization of transcripts identified by FLAIR. A. Structural category distribution for de novo identified transcripts from FLAIR (for ONT libraries) or StringTie (for Illumina libraries), compared to the set of annotated transcripts using SQANTI. The number above each bar represents the number of assembled transcripts. The structural category of a transcript indicates its relation to the closest annotated transcript. B. Number of exons in each transcript identified by FLAIR/StringTie, stratified by the relation to the annotated transcripts (represented by the assigned structural category). C. Length distribution of transcripts identified by FLAIR/StringTie, stratified by the relation to the annotated transcripts (represented by the assigned structural category). The *_ILMNjunc* suffix indicates that FLAIR was supplied with the junctions observed in the Illumina samples. The center line represents the median; hinges represent first and third quartiles; whiskers the most extreme values within 1.5 interquartile range from the box. Source data for panel B are provided as a Source Data file.

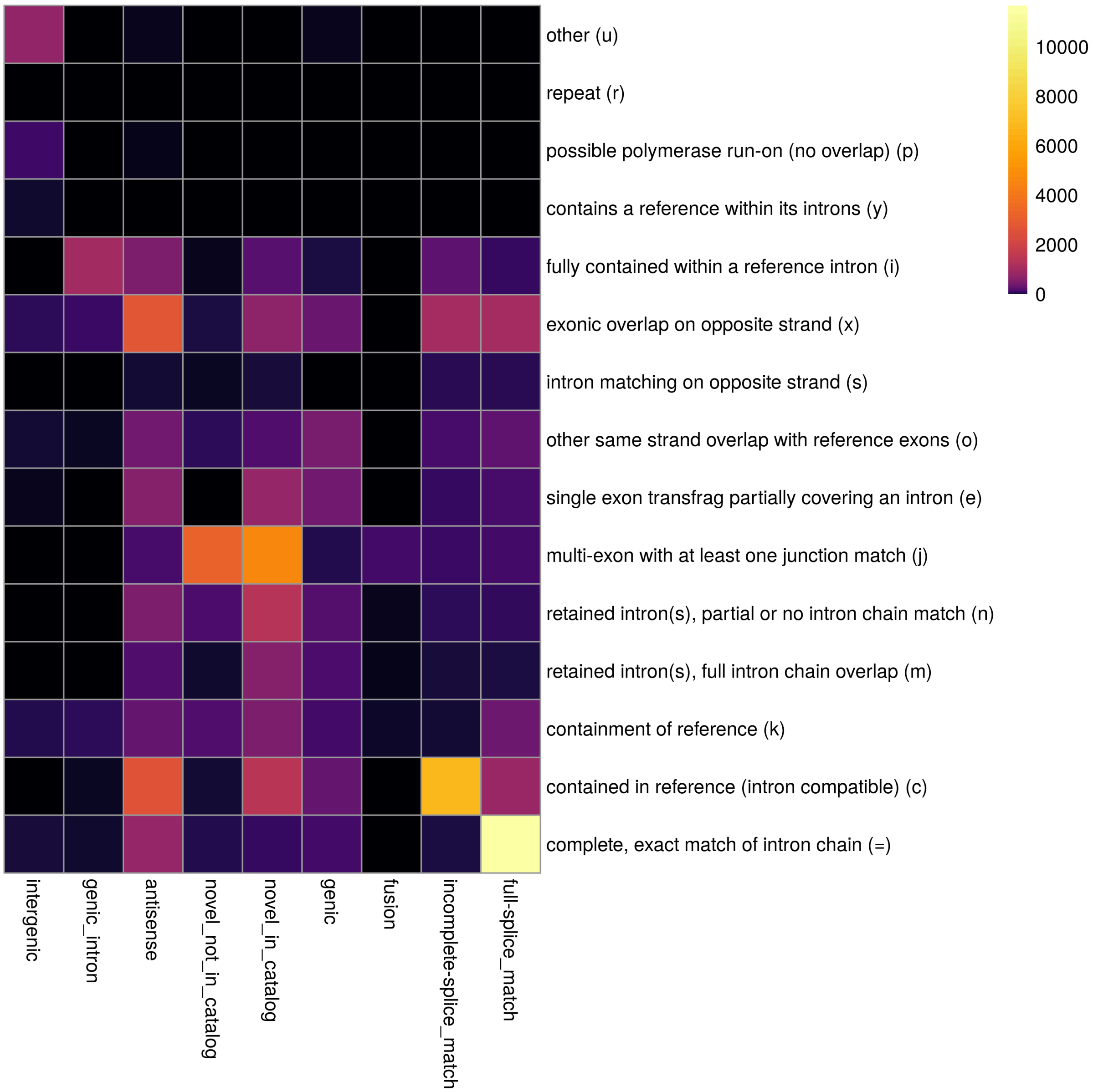

Supplementary Figure 25: Comparison of classification of FLAIR transcripts obtained by SQANTI structural categories (columns) and gffcompare class codes (rows), across all ONT data sets. The color of a square corresponds to the number of FLAIR transcripts annotated to each combination of structural category/class code. Source data are provided as a Source Data file.

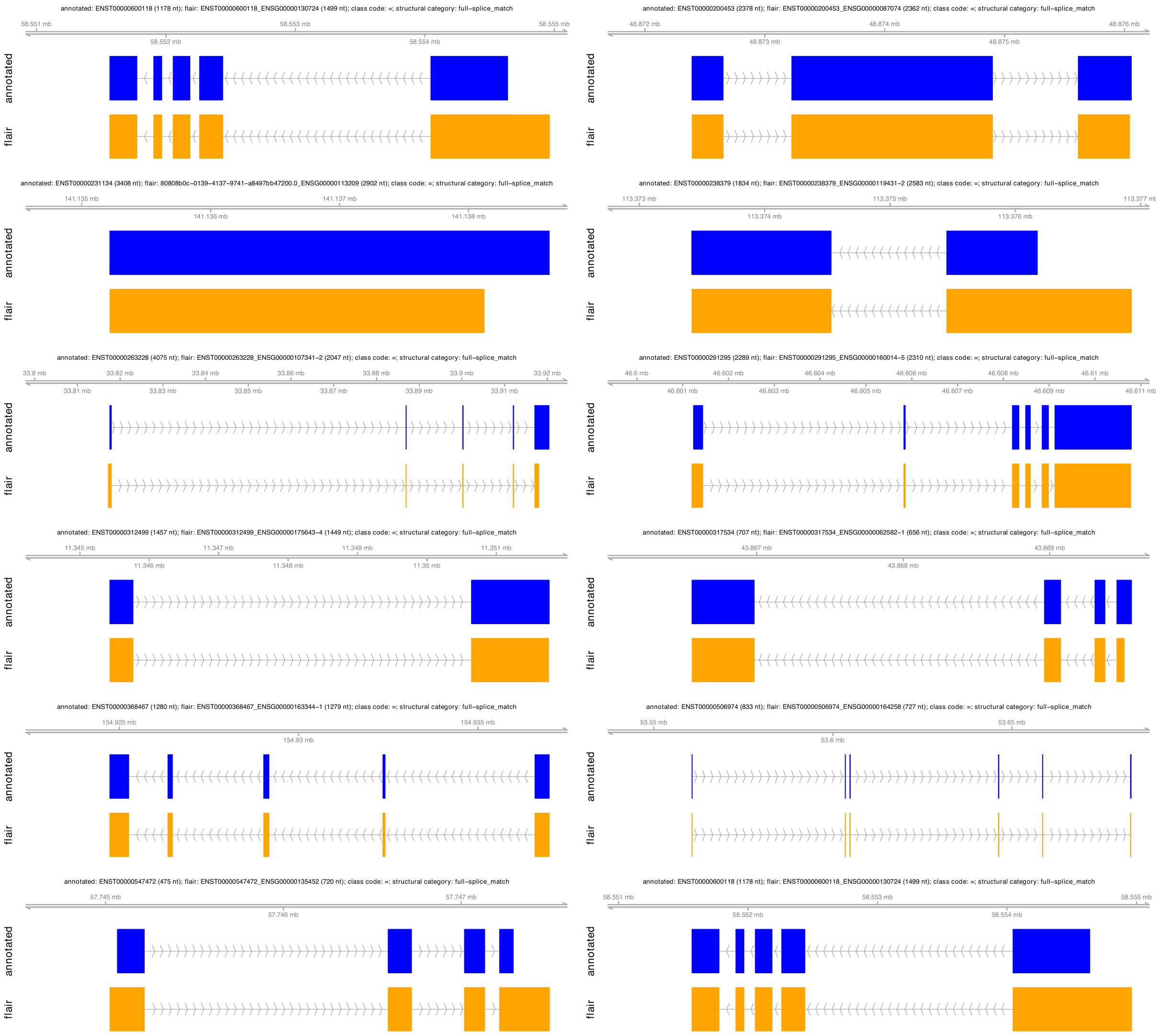

Supplementary Figure 26: Examples of transcripts identified by FLAIR in the ONT-RNA001-HAP data set, with a complete, exact match to the intron chain of an annotated transcript (class code '=' from gffcompare, structural category 'full-splice_match' from SQANTI).
