# Supplementary material for: A comprehensive examination of Nanopore native RNA sequencing for characterization of complex transcriptomes
Source: Nat Commun. 2019 Jul 31;10:3359. doi: 10.1038/s41467-019-11272-z (PMC6668388; doi:10.1038/s41467-019-11272-z)
Supplement: Supplementary file 2 — Reporting Summary [file 41467_2019_11272_MOESM2_ESM.pdf]

nature research

Corresponding author(s): Charlotte Soneson, Mark D Robinson & Shobbir Hussain

Last updated by author(s): June 29, 2019

Reporting Summary

Nature Research wishes to improve the reproducibility of the work that we publish. This form provides structure for consistency and transparency in reporting. For further information on Nature Research policies, see Authors & Referees and the Editorial Policy Checklist.

Statistics

For all statistical analyses, confirm that the following items are present in the figure legend, table legend, main text, or Methods section.

n/a	Confirmed	
☐	☒	The exact sample size (*n*) for each experimental group/condition, given as a discrete number and unit of measurement
☐	☒	A statement on whether measurements were taken from distinct samples or whether the same sample was measured repeatedly
☒	☐	The statistical test(s) used AND whether they are one- or two-sided<br>*Only common tests should be described solely by name; describe more complex techniques in the Methods section.*
☒	☐	A description of all covariates tested
☒	☐	A description of any assumptions or corrections, such as tests of normality and adjustment for multiple comparisons
☒	☐	A full description of the statistical parameters including central tendency (e.g. means) or other basic estimates (e.g. regression coefficient) AND variation (e.g. standard deviation) or associated estimates of uncertainty (e.g. confidence intervals)
☒	☐	For null hypothesis testing, the test statistic (e.g. *F*, *t*, *r*) with confidence intervals, effect sizes, degrees of freedom and *P* value noted<br>*Give P values as exact values whenever suitable.*
☒	☐	For Bayesian analysis, information on the choice of priors and Markov chain Monte Carlo settings
☒	☐	For hierarchical and complex designs, identification of the appropriate level for tests and full reporting of outcomes
☐	☒	Estimates of effect sizes (e.g. Cohen's *d*, Pearson's *r*), indicating how they were calculated

Our web collection on statistics for biologists contains articles on many of the points above.

Software and code

Policy information about availability of computer code

Data collection	Albacore v1.2.6+v2.1.0
Data analysis	All code used for the analysis is available at https://github.com/csoneson/NativeRNAseqComplexTranscriptome<br>Software used: minimap2 v2.12, samtools v1.6, bedtools v2.27.0, bbmap v38.02, RSeQC v2.6.5, Salmon v0.11.0, subread v1.6.0, Wub, FLAIR, TrimGalore! v0.4.4, cutadapt v1.13, STAR v2.5.1b, StringTie v1.3.3b, HISAT2 v2.1.0, R v3.5, Nanopolish, SQANTI v1.2<br>Main R packages: GenomicAlignments v1.32.0, tximport v1.8.0, ggplot2 v3.0.0, cowplot v0.9.2, tailfindr v0.1.0

For manuscripts utilizing custom algorithms or software that are central to the research but not yet described in published literature, software must be made available to editors/reviewers. We strongly encourage code deposition in a community repository (e.g. GitHub). See the Nature Research guidelines for submitting code & software for further information.

Data

Policy information about availability of data

All manuscripts must include a data availability statement. This statement should provide the following information, where applicable:

- Accession codes, unique identifiers, or web links for publicly available datasets
- A list of figures that have associated raw data
- A description of any restrictions on data availability

The raw sequence files have been uploaded to ArrayExpress under accession numbers E-MTAB-7757 [https://www.ebi.ac.uk/arrayexpress/experiments/E-MTAB-7757/] (Illumina) and E-MTAB-7778 [https://www.ebi.ac.uk/arrayexpress/experiments/E-MTAB-7778/] (ONT). The source data underlying Figs 2a-c, 4a-e, 5a-b, 6a-b and Supplementary Figs 1a, 4a-c, 7a-b, 14a-b, 15, 17a-b, 18a-b, 19a-b, 20a-b, 21a-b, 22a-b, 23a-b, 24b, 25 are provided as a Source Data file.

Field-specific reporting

Please select the one below that is the best fit for your research. If you are not sure, read the appropriate sections before making your selection.

☒ Life sciences ☐ Behavioural & social sciences ☐ Ecological, evolutionary & environmental sciences

For a reference copy of the document with all sections, see nature.com/documents/nr-reporting-summary-flat.pdf

Life sciences study design

All studies must disclose on these points even when the disclosure is negative.

Sample size	No sample-size calculations were done, since no statistical tests were performed.
Data exclusions	No samples were excluded from the included data sets.
Replication	Each data set contains at least two replicates.
Randomization	No experimental groups were used.
Blinding	No experimental groups were used.

Reporting for specific materials, systems and methods

We require information from authors about some types of materials, experimental systems and methods used in many studies. Here, indicate whether each material, system or method listed is relevant to your study. If you are not sure if a list item applies to your research, read the appropriate section before selecting a response.

Materials & experimental systems

n/a	Involved in the study	
☒	☐	Antibodies
☐	☒	Eukaryotic cell lines
☒	☐	Palaeontology
☒	☐	Animals and other organisms
☒	☐	Human research participants
☒	☐	Clinical data

Methods

n/a	Involved in the study	
☒	☐	ChIP-seq
☒	☐	Flow cytometry
☒	☐	MRI-based neuroimaging

Eukaryotic cell lines

Policy information about cell lines

Cell line source(s)	HEK293 cells were obtained directly from ATCC. HAP1 cells were obtained directly from Horizon Discovery.
Authentication	None of the cell lines used were authenticated in our own laboratory.
Mycoplasma contamination	All cell lines used tested negative for mycoplasma in our own laboratory.
Commonly misidentified lines (See ICLAC register)	None used.
